# Idiopathic eosinophilic parotitis in an eight-year-old boy: a case report

**DOI:** 10.1186/1752-1947-5-385

**Published:** 2011-08-16

**Authors:** Franco Frati, Rachele Boccardo, Silvia Scurati, Matteo Gelardi, Cristoforo Incorvaia

**Affiliations:** 1 Pediatrics, University Department of Medical and Surgical Specialties and Public Health, Perugia, Italy; 2 Scientific Department, Stallergenes, Milan, Italy; 3 Department of Ophthalmology and Otorhinolaryngology, University of Bari, Bari, Italy; 4 Allergy/Pulmonary Rehabilitation, ICP Hospital, Milan, Italy

## Abstract

**Introduction:**

A number of medical conditions, some of them recently reported, are associated with an increased production of eosinophils. We report the first case of eosinophilic parotitis in the literature.

**Case presentation:**

The patient was an eight-year-old Caucasian boy who presented with a two-year history of recurring acute parotitis with no fever. He had had a total of five episodes with no response to antibiotics, but remission had been achieved with oral corticosteroid therapy. We performed allergy tests for inhalant and food allergens and for haptens, but the results were all negative. The results of echography ruled out sialodochitis. Instead, a swab from the parotid duct led to the detection of a high number of eosinophils.

**Conclusions:**

This report is first in the literature to describe a case of eosinophilic parotitis, and we suggest that a cytological assessment, which is quite simple yet rarely used by physicians, be performed when patients with parotitis of uncertain origin are under evaluation.

## Introduction

A number of medical conditions are associated with increased production of eosinophils. A few of the most well known are eosinophilic pneumonia [1], eosinophilic bronchitis [2], and non-allergic rhinitis with eosinophilia syndrome nares and related disorders [3], but new entities such as eosinophilic esophagitis [4] and others are being added to this list. Very recently, a case of eosinophilic sialodochitis, that is, an inflammation of the parotid salivary duct was reported [5]. In this report, we describe the first case of eosinophilic parotitis in the literature. Parotitis is an inflammation of the parotid salivary gland that can be acute or chronic with acute exacerbations. Concerning the etiology, viral parotitis is more common than bacterial parotitis, and mumps is the most common viral cause of parotitis [6]. The diagnosis is made on the basis of the presence of firm, erythematous swelling in the pre- and post-auricular areas, intense local pain and tenderness, and high fever and chills. Later, massive swelling of the neck and respiratory obstruction may occur. Microbiological data may be obtained by collecting specimens by aspiration from the parotid duct orifice or, when this is not feasible, by obtaining a tissue swab from the papilla of the parotid gland, which is located opposite the second upper molar teeth. In addition, ultrasound imaging or sialography can be used to detect altered morphology, especially in patients with chronic diseases.

### Case presentation

**Figure 1:**
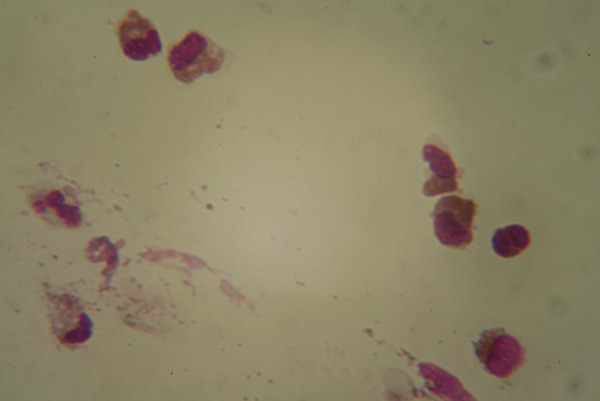
**Microscopic scan showing the material obtained from a swab of the parotid duct**.

Our patient was an eight-year-old Caucasian boy with no family history of atopy who had been in good health until the age of six years, when he started to have recurring episodes of acute parotitis with no fever, amounting to a total of five episodes, and no response to antibiotics, but remission was achieved with oral corticosteroid therapy. We performed allergy tests, including skin prick tests with a standard panel of allergen extracts (Stallergenes, Milan, Italy), patch tests for allergens and haptens, and a radioallergosorbent test for inhalant and food allergens, all of which produced negative results, and echography ruled out sialodochitis. His blood examination, which included tests for anti-nuclear antibodies and anti-DNA antibodies for the assessment of autoimmunity, revealed only a high level of amylase, but a tissue swab taken from the parotid duct allowed us to detect a high number of eosinophils (Figure 1).

## Conclusions

This report describes the first case of eosinophilic parotitis in the published literature, and we suggest that a cytological assessment, which is quite simple though rarely used by physicians, be performed when evaluating patients with parotitis of uncertain origin. Management is based on oral corticosteroid therapy.

## Consent

Written informed consent was obtained from patient's parents for the publication of this case report and any accompanying images. A copy of the written consent is available for review by the Editor-in-Chief of this journal.

## Competing interests

The authors declare that they have no competing interests.

## Authors' contributions

FF and SS analyzed and interpreted the patient data and wrote the manuscript. MG performed the cytological analysis. RB performed the allergy tests. CI was a major contributor to writing the manuscript. All the authors read and approved the final manuscript.
